# Formulation of a Medical Food Cocktail for Alzheimer's Disease: Beneficial Effects on Cognition and Neuropathology in a Mouse Model of the Disease

**DOI:** 10.1371/journal.pone.0014015

**Published:** 2010-11-17

**Authors:** Anna Parachikova, Kim N. Green, Curt Hendrix, Frank M. LaFerla

**Affiliations:** 1 Department of Neurobiology and Behavior, Institute for Memory Impairments and Neurological Disorders, University of California Irvine, Irvine, California, United States of America; 2 Akeso Health Sciences L.L.C., Westlake Village, California, United States of America; University of North Dakota, United States of America

## Abstract

**Background:**

Dietary supplements have been extensively studied for their beneficial effects on cognition and AD neuropathology. The current study examines the effect of a medical food cocktail consisting of the dietary supplements curcumin, piperine, epigallocatechin gallate, α-lipoic acid, N-acetylcysteine, B vitaminsvitamin C, and folate on cognitive functioning and the AD hallmark features and amyloid-beta (Aβ) in the Tg2576 mouse model of the disease.

**Principal Findings:**

The study found that administering the medical food cocktail for 6 months improved cortical- and hippocampal- dependent learning in the transgenic mice, rendering their performance indistinguishable from non-transgenic controls. Coinciding with this improvement in learning and memory, we found that treatment resulted in decreased soluble Aβ, including Aβ oligomers, previously found to be linked to cognitive functioning.

**Conclusion:**

In conclusion, the current study demonstrates that combination diet consisting of natural dietary supplements improves cognitive functioning while decreasing AD neuropathology and may thus represent a safe, natural treatment for AD.

## Introduction

Alzheimer's disease (AD) is a disease of the elderly marked by progressive loss of cognitive functioning. Neuropathologically, AD is characterized by the accumulation of beta-amyloid (Aβ) protein to form plaques and tau phosphorylation resulting in tangle formation. AD is primarily an idiopathic disease with the exception of some rare (<5%) early onset autosomal-dominant familial cases [1]. Dementia is best correlated to synaptic and neuronal loss, rather than directly to pathological burden, and so much interest has been focused on understanding the pathways that lead firstly to the formation of pathology, and then from pathology to synaptic damage, loss and then neuronal death. Strong genetic evidence suggests that it is the aberrant accumulation of Aβ which lies upstream, and that it is this accumulation which leads to downstream pathologies such as tangle formation [2], extensive inflammation [3], oxidative damage to lipids, proteins and DNA [4], and glycation of proteins [5]. Recent studies have highlighted the importance of soluble oligomeric species of Aβ, in particular the 56 kD molecule Aβ*56, in learning and memory [6].

The cause of sporadic AD remains poorly understood but a number of risk factors have been identified, and include many lifestyle and dietary choices, in addition to genetic susceptibility genes, such as the presence of the apoE4 allele [7]. With the identification of these risk factors many studies have focused on understanding how disease progression is impacted using transgenic mouse models of the disease. For example, we have recently shown, using the triple transgenic (3xTg-AD) mouse model of the disease, that stress hormones [8], or nicotine [9] intake increase the severity of AD pathologies, whereas increased dietary intake of omega-3 fatty acids [10], nicotinamide [11], or increased cognitive stimulation [12], can protect against formation of AD pathologies. Dietary intake is particularly important, to general well being, as well as to progression into AD dementia, as highlighted by us and many other groups. Given the complexity and widespread distribution of the pathology, multi-faceted approaches will be required to effectively treat or prevent AD. To that end, we formulated a medical food cocktail comprising agents that are known to reduce Aβ production, but that also show potent anti-oxidant and anti-inflammatory properties, or reduce glycation of proteins. All of these processes have been implicated in AD, and likely contribute to the synaptic and neuronal loss seen in the diseased brain. Previous work has shown that our individual medical food cocktail ingredients have beneficial effects on APP processing, have anti-oxidant, anti-inflammatory or anti-glycation properties. One of the primary components is curcurmin, a polyphenol that comprises the active ingredient of the spice turmeric. Curcurmin is known for its strong anti-oxidant and anti-inflammatory properties, long history of safe use, and low side-effect profile [13], [14]. It has previously been shown to decreased levels of oxidized proteins and interleukin-1 beta in an AD transgenic mice, in addition to a 43–50% decrease in insoluble Aβ, soluble Aβ and amyloid plaque burden [13]. Alongside these results, curcurmin has been shown to inhibit both the formation and growth of Aβ fibrils from Aβ in a dose-dependent manner [15]. To increase the bioavailability of curcumin, and also EGCG, we also included piperine, which is a component of the spice black pepper [16], [17]. Piperine also exhibits significant antioxidant activity of its own, as well as significant chemopreventative and immunomodulary effects [18], [19], [20]. EGCG is a polyphenol that is an active ingredient of green tea. It exhibits potent antioxidant and anti-inflammatory properties as well as confers neuroprotection in AD mouse models [21].

α-Lipoic Acid is a naturally occurring disulfide molecule with antioxidant and anti-inflammatory properties. In a small study of elderly patients with dementia, dietary supplementation with α-lipoic acid stabilized cognitive function, as evidenced by no change in score on 2 neuropsychological tests over more than a 10-month study period [22]. N-Acetylcysteine is an antioxidant, which has been given to AD patients who then demonstrated greater cognitive function than the placebo group after both 3 and 6 months of treatment [23]. Together these ingredients, combined with B-vitamin's (B1, B6, B12 and Folate) and the antioxidants Vitamin C and E provide protection against oxidative damage, which is known to occur in the AD brain.

The medical food cocktail contains the standardized herbal extracts, vitamins and vitamin metabolites, and minerals (Table 1). Together we hypothesized that these ingredients would work synergistically to counteract many of the pathways that contribute to dementia in AD, in order to formulate a safe, natural treatment for AD.

**Table 1:** Components of high and low nutrient combination diets added to AIN-17 rodent chow.

	Active Ingredient Concentration (%)	High Concentration Diet (mg/kg chow)	Consumption per mouse – High (mg/kg body wt/day)	Low Concentration Diet (mg/kg chow)	Consumption per mouse – Low (mg/kg body wt/day)
**Curcumin**	36.58	202.55	50.64	67.52	16.88
**EGCG**	18.29	101.28	25.32	33.76	8.44
**N-Acetylcysteine**	15.26	84.48	21.12	28.16	7.04
**Vitamin B6**	3.72	20.59	5.15	6.86	1.72
**R-α-Lipoic Acid**	9.15	50.69	12.67	16.90	4.23
**Vitamin B1 (benfotiamine)**	3.04	16.83	4.21	5.61	1.40
**Vitamin E** **Succinate**	11.80	65.34	16.34	21.78	5.45
**Vitamin B12**	0.0073	0.041	0.01	0.014	0.003
**Folic Acid**	0.024	0.13	0.033	0.044	0.011
**Vitamin C**	0.30	1.67	0.42	0.56	0.14
**Piperine**	1.84	10.16	2.54	3.39	0.85
**Total**	**100.00**	**553.75**	**138.44**	**184.60**	**46.15**

## Results

To investigate the effects of our medical food cocktail treatment on cognition and AD pathology, we treated 6-month-old Tg2576 and non-transgenic (NonTg) mice with either 1) low dose diet (184 mg/kg; n = 10), 2) high dose diet (553 mg/kg; n = 10) or 3) control diet (n = 10) for a period of 6 months. The well-characterized Tg2576 mouse model of AD exhibits age related accumulation of Aβ plaque pathology starting at 12 months as well as behavioral deficits evident as early as 3 months of age [24]. Following 6 months of treatment, animals were tested on behavioral tasks to examine primarily hippocampus and cortex dependent memory. Treatment continued throughout the behavioral tasks.

### Medical food cocktail treatment restores memory deficits in the Tg2576 mouse model of AD

**Figure 1:**
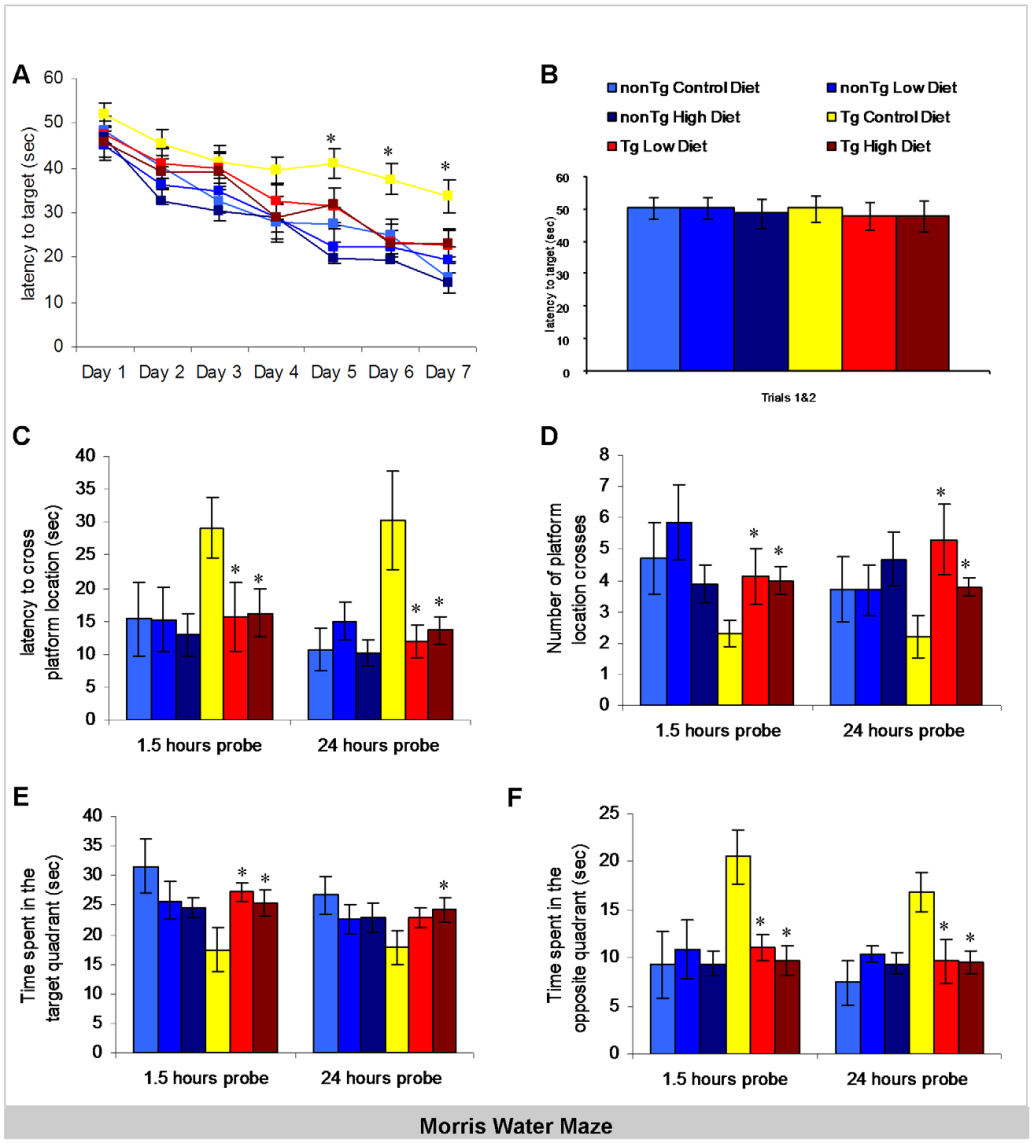
Prevention of hippocampal spatial memory deficits with medical food cocktail diet in Tg2576 mice. Medical food cocktail was given to Tg2576 and non-transgenic control (nonTg) mice at 6 months of age, at a low and high (3x higher) dose. After 5 months of treatment, mice were tested for cognitive functioning on hippocampal and cortical dependent tasks. Mice were trained and tested on the spatial memory version of the Morris water maze (MWM; n = 10 per group). A) Acquisition curves shown for the 7 days of training on the MWM. Non-transgenic mice perform better as compared to Tg2576 starting day 2 of training (Genotype main effect day 2 F(1,45) 3.586, p = 0.0599, day 3 F(1, 45) 4.611 p = 0.0332, day 4 F(1, 45) 4.832, p = 0.0519, day 5 F(1, 45) 13.812, p = 0.003, day 6 F(1, 45) 4.021, p = 0.0465 and day 7 F(1, 45) 15.821, p = 0.0001). NonTg mice on the control diet perform significantly better on day 7 as compared to day 1 of training (p<0.0001). Tg2576 mice on the control diet also exhibited learning during the acquisition phase of the test (day 1 compared to day 7 p<0.05) but were unable to reach the 25 sec criterion after 7 days of training. Medical food cocktail improved the spatial learning of Tg2576 during training (low diet day 5 p<0.05, day 6 p<0.01, day 7 p = 0.1034; high diet day 5 p<0.01, day 6 p<0.001, day 7 p<0.01) with mice reaching criterion by day 7 of training. B) All mice started at the same level as shown by the average of trials 1 and 2 on the 1^st^ day of training (Genotype main effect F(1, 104) 0.094, p = 0.9107, treatment main effect F(2, 104) 0.233, p = 0.7928). C–F) Mice were given a memory probe with the platform removed at 1.5-h or 24-h following the last training trial. C) Tg2576 mice took significantly longer to reach the platform location as compared to non-transgenic mice on the control diet (p<0.05 for both 1.5 and 24-hour probe trials). Tg2576 mice treated with medical food cocktail exhibited significantly decreased latencies to cross the platform location compared to vehicle-treated mice (p<0.05 for both 1.5 and 24-hour probe trials for both low and high diet treatments). D) The deficits in memory were also evident in the number of crosses of Tg2576 as compared to the control diet treated non-transgenic mice (p<0. 05 at both 1.5 and 24 hours). Tg2576 mice treated with medical food cocktail made significantly more platform crosses at both short- and long-term probes than vehicle-treated mice (low diet (p<0.05 at both 1.5 and 24 hours; high diet p<0.01 at 1.5 and p<0.05 at 24 hours). E) Control diet treated Tg2576 mice also spent less time in the target quadrant (p<0.05 for both 1.5 and 24-hour probe trials). Tg2576 mice treated with medical food cocktail spent significantly more time in the target quadrant than vehicle-treated transgenic mice (low diet (p<0.05 at 1.5 hours; high diet p<0.05 at both 1.5 and 24 hours). F) In support of the target quadrant data, time spent in the opposite quadrant was significantly more for Tg2576 as compared to non-transgenic mice on the control diet (p<0.01 for both 1.5 and 24-hour probe trials). Tg2576 mice treated with medical food cocktail spent significantly less time in the opposite quadrant than vehicle-treated mice (low diet (p<0.01 at 1.5 and p<0.05 at 24 hours; high diet p<0.01 at both 1.5 and 24 hours). Error bars indicate SEM. * indicates significance for control Tg2576 mice vs. medical food cocktail treated Tg2576 mice.

Morris water maze is a spatial task found to be primarily dependent on the hippocampus [25]. The MWM protocol used was adapted from [26]. Tg2576 and NonTg mice treated with 1) low dose medical food cocktail, 2) high dose medical food cocktail or 3) control diet were tested. Mice were first trained on the MWM to learn the location of a submerged platform for 4 trials per day until criterion was reached (escape latency <25 s). At the start of the test, all groups performed identically as assessed by their performance on trials 1 and 2 on day 1 depicted in Figure 1B. The average latency to the target platform for all groups during the 7 days of training is represented in Figure 1A. As previously demonstrated NonTg on the control diet performed better on the acquisition phase of the test as compared to age-matched Tg2576 mice. NonTg mice on the control diet demonstrated learning of the behavioral task and performed significantly better on the last as compared to the first day of training. Tg2576 mice on the control diet also exhibited learning during training but were unable to reach criterion after 7 days of training. Notably, Tg2576 mice treated with either low or high doses of the medical food cocktail diets were undistinguishable from NonTg mice, showing that their spatial learning abilities were restored to NonTg levels. Treated Tg2576 mice exhibited improvement in escape latency as compared to Tg2576 on the control diet. 1.5- and 24-hours following the 7 days of training, animals were exposed to probe trial testing sessions, to reflect short-term and long-term memory respectively. During testing, the hidden platform is removed and a number of parameters are measured that are known to reflect aspects of primarily hippocampal-dependent memory. Examining the latency to the target location at 1.5- and 24-hours following training, we find again that transgenic mice on the control diet perform significantly worse than NonTg mice, and that treatment with the medical food cocktail (either low or high dose) significantly improves memory at both time points in the transgenic mice (Figure 1C). Analysis of the number of platform crosses revealed that Tg2576 mice on the control diet cross the region where the platform was located significantly less than NonTg mice. In contrast, medical food cocktail diet treatments in the Tg2576 mice results in increased number of platform crosses (both high or low doses of the medical food cocktail), compared to untreated Tg2576 mice. In addition we also measured the amount of time animals spent in the target quadrant as compared to the opposite quadrant of where the platform used to be located (Figure 1E, F). Notably, these results show that the medical food cocktail treatment restores both acquisition and memory deficits in Tg2576 mice back to non-transgenic levels.

**Figure 2:**
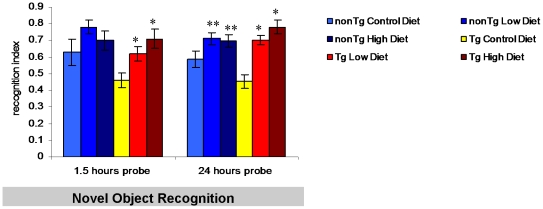
Prevention of cortical memory deficits with medical food cocktail diet in Tg2576 mice. We evaluated treated and untreated Tg2576 and nonTg mice in performance of the primarily perirhinal cortex-dependent contextual task, novel object recognition. Tg2576 mice treated with medical food cocktail had significantly improved recognition indexes at both the 1.5- and 24-h probe trials, compared to vehicle Tg2576 mice (1.5 hour probe trial (low diet p<0.05, high diet p<0.01; 24 hour probe trial (p<0.01 for both low and high combination diets). Notably, nonTg mice treated with medical food cocktail showed improved recognition indexes at the 24-h probe compared to vehicle nonTg mice (p<0.05 for both low and high combination diets). Error bars indicate SEM. * indicates significance for control Tg2576 mice vs. medical food cocktail treated Tg2576 mice, ** indicates significance for control nonTg mice vs. medical food cocktail nonTg mice.

Novel object recognition is based on animals' inherent preference to explore a novel object more than a familiar object. Animals are exposed to two identical objects and 1.5 and 24 hours later they are presented with one familial and one novel object. The amount of time animals spend exploring the novel object is noted and the recognition index is generated. NonTg mice spent more time exploring the novel object as indicated by a recognition index of >0.5 (Figure 2). In contrast, Tg2576 mice on the control diet spent approximately the same amount of time exploring the novel and the familiar object, suggestive of memory deficits. On both the 1.5- and 24-hour retention tests, we found that medical food cocktail treated Tg2576 mice performed significantly better than Tg2576 mice on the control diet and spent more time with the novel object (Figure 2), performing at a level similar to, or even exceeding, non transgenic mice which do not develop AD pathology. Notably, NonTg mice treated with the medical food cocktail also performed better than NonTg mice on the control diet at the 24 hour retention test, suggesting that this medical food cocktail could improve certain types of memory in non pathological animals.

These behavior data suggest that 6-month treatment of Tg2576 mice with a low or high dose of medical food cocktail diet improved primarily cortex and hippocampus dependent memory.

### Medical food cocktail treatment reduces Aβ levels and oligomerization

**Figure 3:**
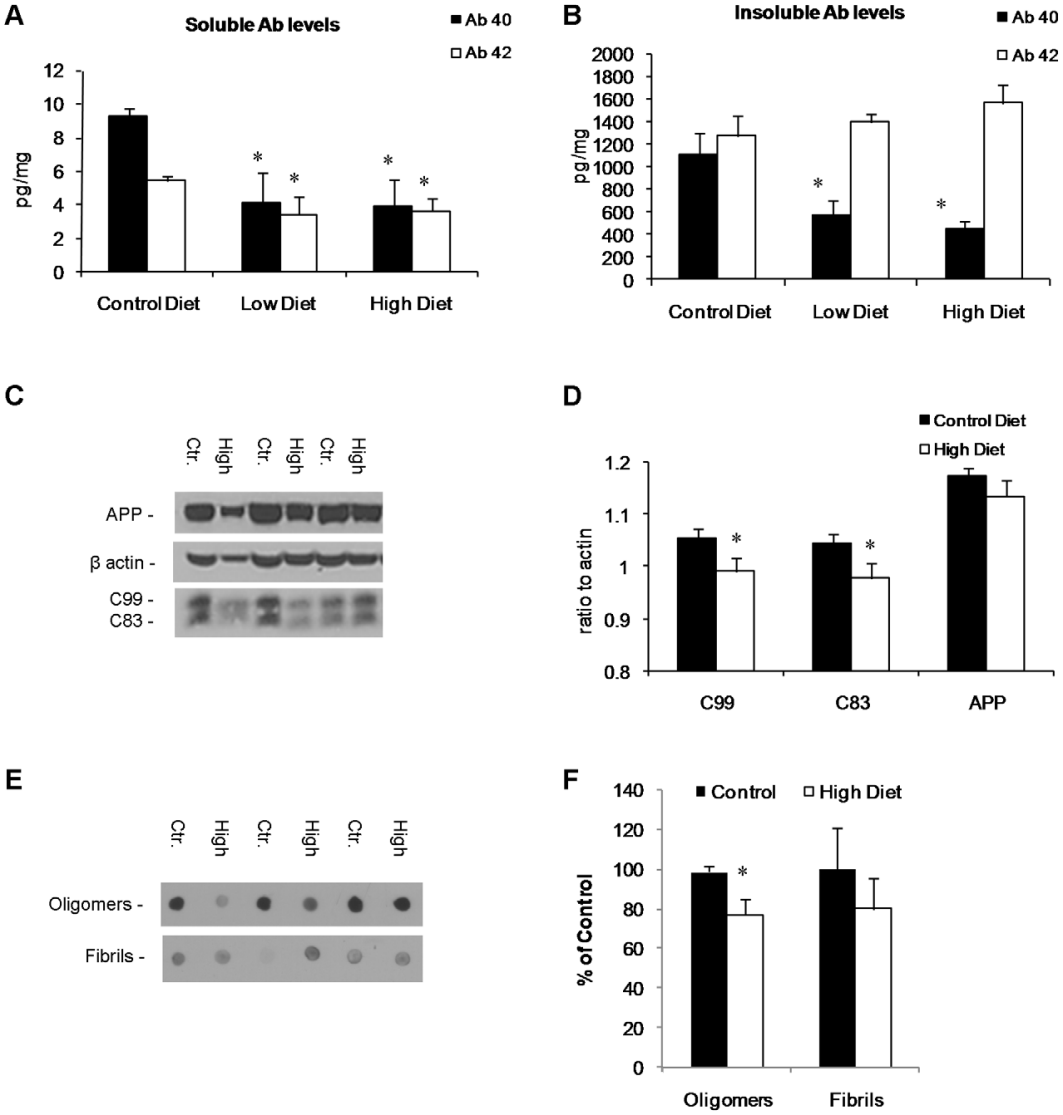
Medical food cocktail diet reduces Aβ levels and decreases aggregation. Soluble (A) and insoluble (B) Aβ_40_ and Aβ_42_ levels were measured from Tg2576 whole brain homogenates from animals treated for 6 months with medical food cocktail or vehicle. A) Soluble Aβ levels were significantly reduced with medical food cocktail diet in Tg2576 mice. B) Insoluble Aβ40 levels were also significantly reduced with medical food cocktail diet in Tg2576 mice. C) Western blot analyses of protein extracts from whole-brain homogenates of Tg2576 mice treated for 6 months with either high dose medical food cocktail or vehicle shown as alternating lanes. Steady state levels of APP were unaffected by treatment, but APP CTF's C83 and C99 were reduced by medical food cocktail treatment. D) Quantification of (C) normalized to β-actin levels as a loading control. E) Dot blot analyses of brain homogenates from Tg2576 mice treated for 6 months with either high dose medical food cocktail or vehicle for Aβ oligomers or Aβ fibrils, using conformation specific antibodies, or western blot analyses for Aβ*56 using 6E10, shown as alternating lanes. Reductions in both Aβ oligomers and Aβ*56 were seen with treatment. F) Quantification of (E). Error bars indicate SEM. * indicates significance (p<0.05) for control Tg2576 mice vs. high dose medical food cocktail treated Tg2576 mice. To assess levels of low molecular weight oligomeric and soluble fibril Aβ species we used the conformation specific antibodies A11 and OC respectively. Dot blot analysis showed a 20% reduction of soluble oligomers in the brains of Tg2576 animals treated with the high combination diet (Figure 2E, F), but no differences in soluble fibrils.

Brain homogenates from both the low and high doses of medical food cocktail treated and control treated Tg2576 mice were assessed for Aβ levels by sandwich ELISA. We found a significant decrease in both Aβ_40_ and the more amyloidogenic Aβ_42_ species in the detergent soluble fraction with both low and high diet (Figure 3A). Analysis of the detergent-insoluble fraction revealed a statistically significant reduction in Aβ_40_ levels with medical food cocktail dietary treatment in the Tg2576 transgenic mice (Figure 3B). Insoluble Aβ_42_ levels were unchanged with treatment.

Next we examined APP processing in treated and untreated Tg2576 mice. APP can be cleaved either via a non-amyloidogenic or an amyloidogenic pathway. The non-amyloidogenic pathway cleaves APP with α-secretase yielding sAPPα and the C83 fragment, which can then be further cleaved via γ-secretase. In contrast, the amyloidogenic pathway results in Aβ production via the sequential cleavage with β- followed by γ-secretase. β-secretase (BACE) cleavage of APP results in the generation of sAPPβ and C99, which is then further cleaved by γ-secretase to yield Aβ. Examining the levels of the APP holoprotein and the cleaved fragments C83 and C99, we found that combination diet treatment resulted in a significant decreased in both C83 and C99 levels (Figure 3C, D).

In addition to examining Aβ levels and APP processing as a result of treatment with combination diet in Tg2576 mice, we further studied the aggregation state of Aβ, using conformation-specific antibodies. We found a significant decrease in the levels of soluble oligomers (∼18–250 kDa species [27]) using A11 antibody, but no difference in soluble fibrils as assessed via OC with low concentration diet (Figure 3E, F). These findings are in line with reduced Aβ in the detergent soluble fraction (Figure 3A). These data suggest that combination diet treatment results in improved cognitive functioning coinciding with reduced levels of soluble Aβ species, including oligomeric species.

## Discussion

The current study provides evidence that a combination diet of dietary supplements, individually known to be beneficial, can not only improve cognitive functioning in a transgenic mouse models of AD but also decreases Aβ levels and oligomerization. As yet there is an unmet need for effective treatments and preventative strategies for AD, and the fastest route to human patients involves the use of either existing medications, or the formulation of known safe remedies. Given that human AD is far more complex than we can effectively model in mice, which develop AD related pathology and cognitive decline but lack extensive neuronal loss, we must formulate treatments that attack not just the symptoms seen in these mice, but also those which we predict will show benefits downstream of pathology that occur in humans. Our formulation here has been designed to alter APP processing through reductions in both Aβ production, as well as aggregation, but also to prevent downstream pathologies such as excessive oxidative damage and inflammation. It is our hope that such a strategy will slow disease progression in humans.

Our rationale is that the individual components of the medical food cocktail work synergistically to produce cognitive and pathological benefits, and together have larger effects than any single component alone. In order to take the step from formulation to human administration we have tested the medical food cocktail in a well-described transgenic mouse model of AD. Serving as a proof of principal we saw cognitive recovery, as well as reduction of Aβ. Our results here show that combination approaches to the treatment of AD are effective in mouse models of AD, and have high translation potential for the human disorder.

## Materials and Methods

### Mice

The study used 6-month old Tg (HuApp695.K670-M671L) 2576 transgenic and age-matched C57Bl6/SJL non-transgenic control mice. Mice were obtained from Jackson Labs and a colony was established. Tg2576 transgenic mice over-express human APP with the double Swedish mutation [28]. Tg2756 and control mice were treated for a period of 6 months with either 1) low dose diet (184 mg/kg; n = 10), 2) high dose diet (553 mg/kg; n = 10) or 3) control diet (n = 10). The contents of the medical food cocktail were supplemented to the standard AIN-17 rodent chow.

After treatment, the animals were sacrificed and the brains removed. The brains were immediately dissected in half along the coronal line and one-half frozen for biochemical analysis and the other half fixed in 4% paraformaldehyde.

### Animal Treatments and Ethics

All rodent experiments were performed in accordance with animal protocols approved by the Institutional Animal Care and Use Committee at the University of California, Irvine (UCI).

### Behavioral Testing

#### Morris water maze

The Morris Water Maze (MWM) is a test for spatial memory (i.e. hippocampus dependent) and cued learning (i.e. non-hippocampal) in rodents. Many studies in the last two decades have used this test as a reliable measure of hippocampal-dependent learning [29], including several transgenic models [28], [30]. All animals were handled briefly on the 4 days prior to each block of training, and all animals were tested on motor (gait and stepping) tasks and given a general health assessment evaluating coat, eye and nose condition, and hind leg clasping. Hidden and cued platform Morris water maze (MWM) training and testing were conducted as described previously [26]. Mice were trained to swim to a 14-cm diameter circular clear Plexiglass platform submerged 1.5 cm beneath the surface of the water. The platform location was selected randomly for each mouse, but was kept constant for each individual mouse throughout training at each age. On each trial, the mouse was placed into the tank at one of four designated start locations and allowed to find and escape onto the platform. If a mouse failed to find the platform within 60 s, it was manually guided to the platform and allowed to remain there for 5 s. After this, each mouse was placed into a holding cage under a warming lamp for 25 s until the start of the next trial.

Retention of the spatial training was assessed 1.5 hr and again 24 hr after the last training trial. Both probe trials consisted of a 60 s free swim in the pool without the platform. Mice were monitored by a camera and all trials were scored during the probe trial and again after the probe trial for verification. There were no significant differences between any genotypes in the swim speeds, degree of thigmotaxis or floating. The parameters measured during the probe trial included (1) initial latency to cross the platform location; (2) number of platform location crosses; and (3) time spent in the quadrant opposite to the target quadrant.

#### Novel object recognition

This task is based on the spontaneous tendency of rodents to explore a novel object more often than a familiar object [31]. Perirhinal cortex lesions and studies of neuronal activation and responses in rats suggest that it is cortical and not hippocampal neurons that are involved in the object recognition task [32], [33]. Hippocampal involvement in this task has also been suggested [34], [35], [36] and this task is widely used to study memory impairments in AD models [37], [38]. For the novel object task, mice were familiarized with an empty open field for a period of 10 minutes. On the following day, mice were subjected to a 5 minute exploration session in the same context with two identical objects (Object A; e.g. two identical balls or two identical dice) placed in symmetrical locations in the open field. 90 minutes and 24 hours later, animals were subjected to a 3 minute retention phase test where they were exposed to one Object A and also to a novel object, Object B (for the 90 min time point) and Object C (for the 24 h time point) placed in the same, symmetrical locations in the open field.

The time spent exploring the familiar object and the novel object were calculated where exploration equals touching the object with nose or paws, or sniffing within 1.5 cm of the object. Time spent with the novel object as compared to time spent with both objects was used as memory index.

### Immunoblotting

Protein extracts were prepared from whole brain samples by homogenizing in T-per (Pierce Biotechnology, Rockford, Il, USA) extraction buffer and Complete Mini Protease Inhibitor Tablets (Roche, Indianapolis, IN, USA) followed by high-speed centrifugation at 100,000 g for 1 h. The supernatant was taken as the protein extract. Protein concentrations were determined by the Bradford method. Equal amounts of protein (20 µg–50 µg depending on protein of interest) were separated by SDS/PAGE on a 10% Bis/Tris gel (Invitrogen, Carlsbad, CA, USA), transferred to 0.45 µM nitrocellulose membranes, blocked for 1 hour in 5% (vol/vol) nonfat milk in Tris-buffered saline (pH 7.5) supplemented with 0.2% Tween20, and processed as described. Antibodies and dilutions used in this study include 6E10 (1∶1000 Signet, Dedham, MA, USA), CT20 (1∶5,000; Calbiochem, San Diego, CA, USA) for C99 and C83, and αActin (1∶10,000; Sigma-Aldrich, USA). Quantitative densiometric analyses were performed on digitised images of immunoblots with Scion Image 4.0 (Scion Corporation, Frederick, MD, USA).

### Dot Blot

Ten µg of protein was made up to 10 µl in H_2_0 and pipetted onto 0.45 µM nitrocellulose membrane (Pierce Biotechnology) and allowed to dry. The membrane was blocked for 45 minutes in 5% powder milk in TBS-T and then incubated in A11, or OC (generous gifts from Charlie Glabe, UCI) at 1∶1000 overnight at 4°C. The membrane was then washed 5 times in TBS-T and incubated for 1 hour in HRP goat-anti Rabbit antibody (1∶10000, Sigma-Aldrich). Following an additional 5 washes the membrane was coated with ECL plus (Amersham) and then developed on photographic film. Quantitative densiometric analyses were performed on digitized images of immunoblots using Scion Image 4.0 software (Scion Corporation).

### Aβ ELISA

Aβ_1–40_ and Aβ_1–42_ were measured using a sensitive sandwich ELISA system. Soluble and insoluble Aβ was isolated from whole brain homogenates using T-per Extraction Buffer (Pierce Biotechnology, Rockford, Il, USA) and 70% formic acid (FA) respectively. Soluble fractions were loaded directly onto ELISA plates and FA fractions were diluted 1∶20 in neutralization buffer (1 M Tris base; 0.5 M NaH_4_PO_4_) prior to loading. Secreted Aβ was measured from *in vitro* assays by direct addition of the cell incubated media onto the ELISA plates. MaxiSorp immunoplates (Nunc, Rochester, NY, USA) were coated with mAB20.1 (William Van Nostrand, Stony Brook University, NY) antibody at a concentration of 25 µg/ml in Coating Buffer (0.1 M NaCO_3_ buffer, pH 9.6), and blocked with 3% BSA. Standards of both Aβ_40_ and _42_ were made in Antigen Capture Buffer (ACB; 20 mM NaH_2_PO_4_; 2 mM EDTA, 0.4 M NaCl; 0.5 g CHAPS; 1% BSA, pH 7.0), and loaded onto ELISA plates in duplicate. Samples were then loaded in duplicate and incubated overnight at 4°C. Plates were washed and then probed with either HRP-conjugated anti-Aβ 35-40 (C49, for Aβ_1–40_ (David Cribbs, University of California, Irvine)) or anti-Aβ 35-42 (D32, for Aβ_1–42_ (David Cribbs, University of California, Irvine)) overnight at 4°C. 3,3′,5,5′-tetramethylbenzidine was used as the chromagen, and the reaction stopped by 30% O-phosphoric acid, and read at 450 nm on a Molecular Dynamics plate reader.

### Statistics

Behavioral scores were analyzed using a multifactor or repeated measures ANOVA including genotype or treatment as independent variables, and escape latencies during training and probe trial measures as dependent variables. To dissect complex interactions between factors, post-hoc *Scheffe* tests and Bonferroni corrections were used to determine individual differences between groups. Biochemical data was analyzed using planned Students T-tests. For individual planned comparisons, results were reported as significant only when P<0.05.
